# Prognostic Value of Albumin-to-Fibrinogen Ratio for 28-Day Mortality among Patients with Sepsis from Various Infection Sites

**DOI:** 10.1155/2022/3578528

**Published:** 2022-08-10

**Authors:** Shuai Li, Yawei Shen, Bowen Chang, Nan Wang

**Affiliations:** ^1^Department of Critical Care Medicine, The First Affiliated Hospital of Anhui Medical University, Hefei, Anhui, China; ^2^Anhui Public Health Clinical Center, Hefei, Anhui, China; ^3^The First Affiliated Hospital of USTC, University of Science and Technology of China, Hefei, China

## Abstract

**Purpose:**

This study investigated the prognostic value of the albumin-to-fibrinogen ratio (AFR) in patients with sepsis as a consequence of infection at various sites.

**Methods:**

A total of 300 patients with sepsis caused by various infection sites, who met the diagnostic criteria for sepsis hospitalized in the intensive care unit, were enrolled in this study. The observational endpoint was 28-day mortality. Cox proportional hazard regression analysis was performed to determine the potential prognostic factors for 28-day mortality in these septic patients. Receiver operating characteristic (ROC) curve analysis was used to evaluate and compare the prognostic factors for 28-day mortality.

**Results:**

Of 300 participants, 147 died, corresponding to a 28-day mortality of 49% (147/300). Baseline Acute Physiology and Chronic Health Evaluation (APACHE II) score (hazard ratio (HR) 1.18 (95% confidence interval (CI) 1.07–1.30); *P* < 0.001), baseline lactic acid level (HR 1.27 (95% CI 1.08–1.50); *P* = 0.005), the presence of septic shock (HR 21.44 (95% CI 2.51–182.76); *P* = 0.005), and baseline AFR (HR 0.70 (95% CI 0.62–0.80); *P* < 0.001) were independent prognostic factors for 28-day mortality in patients with sepsis according to multivariate Cox analysis. Baseline AFR was an effective predictor of 28-day mortality, with an area under the ROC curve (AUC) of 0.700, and a specificity and sensitivity of 90.8% and 42.1%, respectively. A low baseline AFR level was associated with increased 28-day sepsis-related mortality. The quadruple index, which included the APACHE II score, lactic acid, septic shock, and AFR, showed a more accurate predictive value for septic patients than the APACHE II score, lactic acid, septic shock, and AFR alone, with an AUC of 0.922, and specificity and sensitivity of 86.9% and 83.6%, respectively. Moreover, the triple index, which included the APACHE II score, lactic acid, and septic shock, showed a significantly lower prognostic value for 28-day mortality compared with the ROC curve of the quadruple index and triple index, with an AUC of 0.877 and specificity and sensitivity of 77.8% and 82.3%, respectively.

**Conclusions:**

The results of this study demonstrate that AFR is an independent protective factor for predicting 28-day mortality in patients with sepsis due to various infection sites. AFR combined with the APACHE II score, lactic acid, and septic shock showed a higher prognostic value for sepsis prognosis.

## 1. Introduction

Sepsis is a syndrome involving systemic multiple organ dysfunction due to infection. It is the most common cause of death and accounts for substantial economic burden to adults hospitalized in intensive care units (ICUs) [1, 2]. Despite advances in medical and scientific research, however, millions of individuals worldwide continue to be affected by sepsis each year, with a mortality rate of ≥25% [3]. Clinical studies have shown that early detection of sepsis and timely targeted treatment of prognostic risk factors can significantly reduce its mortality rate [4, 5]. This emphasizes the need to identify reliable prognostic factors to accurately predict sepsis outcomes and to accelerate the design of individualized treatment strategies. Sepsis involves complex systemic inflammatory network effects, immune dysfunction, and impaired coagulation function and so on [6]. Accurate and effective biomarkers are important for the diagnosis and prognosis of sepsis. Previous studies have reported that C-reactive protein (CRP) and procalcitonin (PCT) are valuable prognostic factors, which are not satisfactory for predicting sepsis-related mortality [7]. Recently, there have been many studies on the relationship between albumin and fibrinogen levels and sepsis prognosis [5, 8].

Accumulating evidence has shown that serum albumin level is closely associated with inflammation and nutrition and is significantly associated with prognosis in patients with sepsis [9–11]. Fibrinogen, another essential inflammatory biomarker of acute-phase inflammation, is a key protein in the coagulation cascade and plays an important role in the prognosis of sepsis, similar to CRP [12, 13]. Studies have shown that the index integrating albumin and fibrinogen, e.g., albumin-to-fibrinogen ratio (AFR), presents not only the status of inflammation and nutrition but also the coagulation function. With improved consistency compared to albumin or fibrinogen alone, AFR could amplify the sensitivity of inflammatory status and is superior in assessing the prognostic value of various diseases [14–16].

However, to our knowledge, few studies have evaluated the role of AFR in patients with sepsis. As such, the present investigation aimed to assess the utility of selected prognostic factors, including AFR, in patients with sepsis from various infection sites.

## 2. Material and Methods

### 2.1. Study Design

This retrospective study was approved by the Ethics Committee of the Affiliated Hospital of Anhui Medical University (Hefei, Anhui province, China). This study initially enrolled eligible patients who were admitted to the ICU of Affiliated Hospital of Anhui Medical University between August 2018 and August 2021. The inclusion criteria were as follows: (1) adults (male and female) > 18 years of old; (2) presence of sepsis according to the definition criteria (Sepsis-3) [1, 17] caused by various foci of infection; and (3) diagnosis of sepsis within 24 h after admission to the ICU. Patients < 18 years of age, who did not complete the 28-day follow-up data, who were pregnant, patients with advanced cancer (patients with solid tumors or hematologic malignancies who failed standard therapy or relapsed, and the presence of radiographic evidence of metastatic disease in solid tumors was required) [18], severe malnutrition (weight loss > 10% within the last 6 months, or >20% after 6 months) [19], coagulopathy (e.g., hemophilia), hepatic dysfunction (e.g., decompensated cirrhosis), and immunodeficiency diseases (e.g., systemic lupus erythematosus), and those receiving glucocorticoid, radiotherapy, chemotherapy, or immunotherapy were excluded. All participants were treated and managed in the ICU according to international guidelines for sepsis and septic shock [4]. The observational endpoint was 28-day mortality. Telephone follow-up was used to assess patients who were discharged from hospital before 28 days.

### 2.2. Definition of Sepsis

Sepsis was diagnosed according to the Third International Consensus Definitions as an acute change in total sequential organ failure assessment (SOFA) score ≥ 2 points consequent to the infection.

### 2.3. Data Collection

Clinical data were collected from the medical records of each enrolled patient. Demographic information included age and gender, baseline vital signs, including body temperature, heart rate, respiratory rate, and mean atrial pressure, and the intervention strategies including mechanical ventilation and continuous renal replacement therapy. Detailed data regarding common comorbidities, including hypertension, diabetes mellitus, chronic lung disease, cardiac disease, and chronic renal disease, were extracted from the database. Sites of infection included the bloodstream and the respiratory, genitourinary, gastrointestinal, hepatobiliary, and central nervous systems, according to the results of microbial culture. Patient with suspected site of infection without clear pathogen was considered an unknown site of infection. In addition, the Acute Physiology and Chronic Health Evaluation (APACHE) II score [20], SOFA score [21], and Glasgow Coma Scale (GCS) [22] score were calculated based on methods reported in the literature. Laboratory investigations included white blood cell (WBC) counts, neutrophils (NEU), lymphocytes (LYM), hemoglobin (Hb), platelets (PLTs), pH, lactic acid, CRP, PCT, creatinine, blood urea nitrogen (BUN), albumin, fibrinogen, and AFR. Baseline vital signs were obtained on the day of sepsis diagnosis. Intervention strategies were administered after sepsis diagnosis. The severity of the sepsis was assessed within 24 h of sepsis diagnosis. Peripheral venous and arterial blood samples and samples from infection sources were collected on the day of sepsis diagnosis and measured by our institution laboratory to obtain laboratory data.

### 2.4. Statistical Analysis

Empower (R) (http://www.empowerstats.com, X&Y Solutions, Inc., Boston, MA, USA) and R (http://www.R-project.org) were used for all statistical analyses. Continuous variables are presented as the mean ± standard error (SE) or the median, and categorical variables are presented as number with percentages. Initially, the Kolmogorov–Smirnov test was used to examine whether the variables were normally distributed. Subsequently, the chi-square test, Fisher exact test, Student *t*-test, and Mann–Whitney *U* test were used for data analysis as appropriate. Univariate and multivariate Cox proportional hazard regression analyses were performed to assess the association between prognostic factors, including AFR, and the prognosis of sepsis. In addition, the value of the abovementioned AFR and the potential prognostic factors in sepsis were evaluated and compared according to the area under the receiver operating characteristic (ROC) curve analysis. After post hoc analysis, the maximum sum of sensitivity and specificity was calculated. Statistical significance was set at *P* value < 0.05.

## 3. Results

### 3.1. Baseline Characteristics and Clinical Outcomes

**Figure 1:**
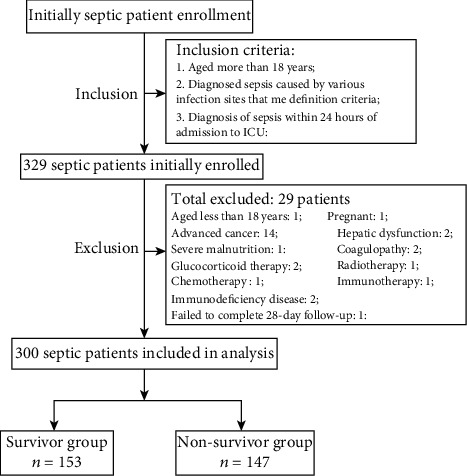
Flow chart of patient selection.

As demonstrated by the flow chart diagram seen in Figure 1. A total of 329 potentially eligible patients with sepsis were initially enrolled in the present study; however, 29 were excluded in accordance with the exclusion criteria. Ultimately, therefore, 300 patients were included in the data analysis. The mean age of the subjects was 62.9 years, and the majority were male (198/300 (66%)). The 28-day mortality of all study participants was 49% (147/300). The most frequent infection sites were the respiratory and genitourinary systems and the bloodstream. Moreover, 87 sepsis patients failed to identify the site of infection. The baseline characteristics of survivors versus non-survivors are summarized in Table 1. No significant differences were noted between the two groups (i.e., survivors versus nonsurvivors) with regard to age and gender distribution. Common comorbidities, including hypertension, diabetes mellitus, cardiac disease, and chronic renal disease, were not remarkably different, except for chronic lung disease between the survivor and nonsurvivor groups. Additionally, baseline vital signs were statistically different in addition to body temperature between the two groups. Moreover, patients with septic shock and those who received mechanical ventilation and continuous renal replacement therapy exhibited higher 28-day mortality rates. Compared with the survivor group, the nonsurvivor group had significantly higher APACHE II and SOFA scores and lower baseline GCS scores. Finally, the site of infection was significantly associated with prognosis among patients with sepsis.

### 3.2. Laboratory Variables

A detailed comparison of laboratory variables between the survivor and nonsurvivor groups is presented in Table 2. In terms of WBC, NEU, LYM, Hb, PLT, CRP, and PCT, there was no obvious correlation with prognosis in either of the two patient groups. The nonsurvivor group was significantly associated with higher levels of lactic acid, BUN, creatinine, and fibrinogen compared with the survivor group. In contrast, patients with sepsis who experienced 28-day mortality exhibited significantly lower levels of albumin, AFR, and pH than those who survived beyond 28 days.

### 3.3. Risk Factors for 28-Day Mortality

**Figure 2:**
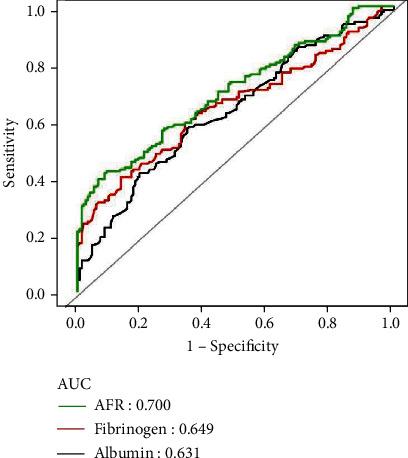
The ROC curve analysis of albumin, fibrinogen, and AFR for the prognostic value of 28-day mortality in patients with sepsis. ROC: receiver operating characteristic; AUC: area under the curve; AFR: albumin-to-fibrinogen ratio;

As shown in Tables 3, 17 potential risk factors (*P* < 0.05) (Tables 1 and 2), age and gender for possible prognostic values for 28-day mortality in the two groups were evaluated using univariate and multivariate Cox proportional hazards regression analyses. Univariate Cox analysis revealed that history of chronic lung disease, the presence of septic shock, use of mechanical ventilation, baseline heart rate, respiratory rate, mean atrial pressure, GCS, APACHE II score, SOFA score, site of infection, pH, lactic acid, BUN, creatinine, albumin, fibrinogen, and AFR were probable valuable prognostic factors in septic patients. More specifically, age, gender and all potential risk factors with a *P* value < 0.1 in the univariate Cox analysis were entered into multivariate Cox regression analysis. However, AFR consists of albumin and fibrinogen. As indicated in Figure 2, the ROC curve analysis was used to investigate which of the two independent components and AFR were more suitable for inclusion in the multivariate Cox regression analysis. AFR revealed higher area under the ROC curve (AUC) value in comparison to fibrinogen (*P* = 0.001), as a probable predictor of 28-day mortality in patients with sepsis. There was a trend toward higher AUC value than albumin, although the difference was not statistically significant (*P* = 0.095). Therefore, AFR was selected for multivariate Cox regression analysis. As shown in Table 3, the results revealed that baseline APACHE II score, baseline lactic acid level, the presence of septic shock, and baseline AFR were independently associated with 28-day mortality in patients with sepsis from various infection sites.

### 3.4. Prognostic Value of Risk Factors, including AFR, for 28-Day Mortality

**Figure 3:**
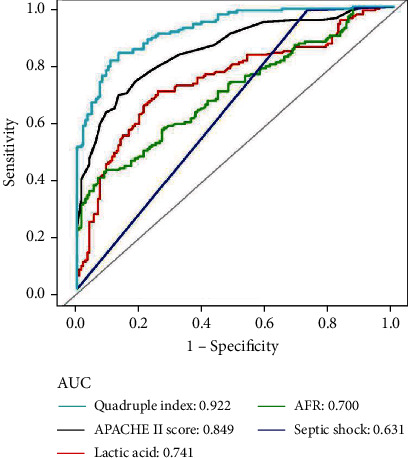
The ROC curve analysis of APACHE II score, lactic acid, septic shock, AFR, and quadruple index for the prognostic value of 28-day mortality in patients with sepsis. ROC: receiver operating characteristic; AUC: area under the curve; APACHE II: Acute Physiology and Chronic Health Evaluation; AFR: albumin-to-fibrinogen ratio; quadruple index includes APACHE II score, lactic acid, septic shock, and AFR;

As shown in Figure 3 and Table 4, the results further demonstrated that baseline AFR was strongly associated with 28-day mortality in patients with sepsis, with an AUC of 0.700 and specificity and sensitivity of 90.8% and 42.1%, respectively. ROC curve analysis revealed that the optimal cut-off value for AFR was 5.86. Moreover, compared with APACHE II score and lactic acid level, ROC curve analysis revealed that AFR had a weak prognostic value for 28-day mortality in patients with sepsis. However, the combined quadruple index, which included the APACHE II score, lactic acid, septic shock, and AFR, was a better predictor of 28-day mortality in patients with sepsis than the APACHE II score, lactic acid, septic shock, and AFR alone, with an AUC of 0.922, and specificity and sensitivity of 86.9% and 83.6%, respectively.

### 3.5. Comparison of Quadruple Index and Triple Index for Predicting 28-Day Mortality

**Figure 4:**
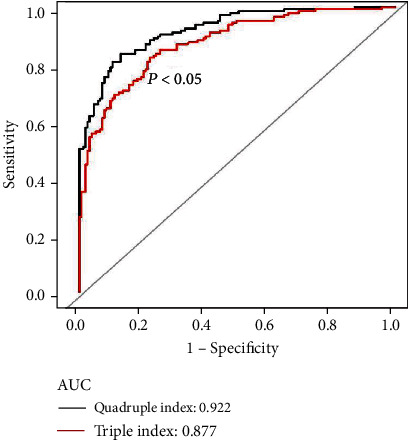
Comparison of the ROC curves of quadruple index and triple index for the prognostic value of 28-day mortality in patients with sepsis. ROC: receiver operating characteristic; AUC: area under the curve; APACHE II: Acute Physiology and Chronic Health Evaluation; AFR: albumin-to-fibrinogen ratio; triple index includes APACHE II score, lactic acid, and septic shock; quadruple index includes APACHE II score, lactic acid, septic shock, and AFR;

As shown in Figure 4 and Table 4, to further validate the prognostic value of baseline AFR for 28-day mortality in patients with sepsis, we analyzed the triple index comprising the APACHE II score, lactic acid, and septic shock, with an AUC of 0.877 and specificity and sensitivity of 77.8% and 82.3%, respectively. Compared with the ROC curve of the quadruple index, the addition of AFR significantly increased the prognostic value of 28-day mortality in patients with sepsis.

## 4. Discussion

The results of this retrospective observational study revealed that a high baseline APACHE II score, a high baseline lactic acid level, the presence of septic shock, and a low baseline AFR were independently associated with a greater risk of 28-day mortality in patients with sepsis caused by various foci of infection. To our knowledge, few studies have examined AFR as an independent prognostic biomarker in patients with sepsis. Moreover, AFR along with the APACHE II score, lactic acid, and septic shock showed a higher prognostic value of 28-day mortality than the APACHE II score, lactic acid, septic shock, and AFR alone in patients with sepsis.

Sepsis is defined as a life-threatening, infection-induced, decompensated host response leading to multiple organ dysfunction [1]. Early recognition of and timely intervention for sepsis are indispensable for improving the survival rate and preventing the transition to septic shock, which has a mortality rate ≥ 40%[3, 23]. Moreover, an increasing number of studies have attempted to explore effective prognostic factors in attempts to reduce sepsis-related mortality [10, 24, 25]. However, prognostic factors have not been completely identified in patients with sepsis.

Our results from multivariate Cox regression analysis demonstrated that baseline APACHE II score, baseline lactic acid level, septic shock, and baseline AFR were independent prognostic factors in septic patients caused by various infection sites. The APACHE II score is extensively used among critically ill patients with sepsis in the ICU, and is accurate for the prognosis, comparable the SOFA score [1, 26]. Our results are also consistent with the apparent correlation between the APACHE II score and prognosis of sepsis. Evidence-based recommendations have demonstrated that a high baseline APACHE II score strongly supports a poorer prognosis of sepsis [27, 28]. However, our study reported that the SOFA score was not an independent prognostic factor for 28-day mortality [14]. A systematic review by Calle et al. concluded that the SOFA score could not assess the accuracy of the prognostic value in patients with suspected infection [29]. In our consideration, the inconsistent conclusions may be due to the different age ranges, infection sites, and severity of sepsis. Further research may be necessary to validate the prognostic value of the SOFA score in septic patients.

In our study, septic shock was also revealed to be an independent prognostic factor in patients with sepsis caused by various infection sites. According to SEPSIS 3.0, septic shock indicates that the exacerbation of sepsis leads to circulatory and cellular metabolism abnormalities and can be diagnosed by clinical features, including sustained hypotension requiring vasopressors and persistent serum lactate level ≥ 2 mmol/L, despite adequate volume resuscitation [1]. Many studies have shown that septic shock is closely associated with higher mortality [3], which is in accordance with our conclusions. However, further investigations are needed to identify other efficient prognostic factors of sepsis due to the lack of septic shock symptoms in the early stages of sepsis.

High blood lactate level is a well-known biomarker associated with poor prognosis among patients with sepsis, which is significantly increased by tissue hypoxia [30]. Our findings provide evidence supporting that a high baseline level of lactic acid is associated with increased mortality in patients with sepsis. An increasing number of studies and sepsis guidelines suggest that lactate levels > 2 mmol/L are more sensitive in predicting prognosis in patients who experience septic shock [4, 31]. A previous study by Casserly et al. concluded that lactate levels > 4 mmol/L were associated with higher 28-day mortality rates [32]. This study is consistent with our best threshold of blood lactate levels from the ROC curve analysis, which may provide a more accurate prognostic valve for 28-day mortality.

Our study also found that AFR, a new biomarker, was strongly associated with 28-day mortality in patients with sepsis caused by various infection sites. AFR consists of albumin and fibrinogen, which are widely used as prognostic factors in patients with sepsis [5, 8]. A previous report by Don et al. indicated that inflammation leads to low serum levels of albumin due to decreased synthesis and increased catabolism. Low albumin levels significantly influence the pharmacokinetics of antimicrobials, resulting in decreased antibacterial effects, except for poor nutritional status and organic function [33]. This may explain why albumin is closely associated with sepsis prognosis. However, study by Godinez-Vidal et al. reported that albumin can be considered a predictor of severity, although not a predictor of mortality [34]. Serum fibrinogen expression is notably increased in patients with a high inflammatory status and can promote the activation of coagulation and aggregation of PLT [16, 35]. Previous studies have shown that dysregulation of fibrinogen expression leads to a poor prognosis of disseminated intravascular coagulation in patients with sepsis [13]. In summary, the levels of albumin or fibrinogen may be not effective prognosis factor in predicting sepsis-related mortality.

AFR, the ratio of albumin to fibrinogen, can be used as a timely indicator of the nutritional status, inflammatory status, and coagulation function of patients [36–38] and is also an effective marker to predict the prognosis of various diseases, including lung cancer, rheumatoid arthritis, and acute kidney injury [16, 36, 39]. The prognosis of sepsis is closely associated with nutritional status, inflammatory status, and coagulation function of patients [4]. These associations may explain the potential relationship between AFR and sepsis prognosis. Tai et al. first reported AFR as an independent prognostic factor in patients with sepsis, in whom sepsis was caused by abdominal infection after surgical treatment [14]. Our study initially evaluated the role of AFR in patients with sepsis caused by various infection sites. Multivariate Cox regression and ROC curve analysis revealed that a low baseline AFR was significantly associated with increased 28-day mortality in patients with sepsis. While, compared with the ROC curve of the combined index of quadruple and triple, the importance of AFR for prognostic value in patients with sepsis was further validated. Consequently, individualized therapies can be designed according to the baseline AFR, to improve the prognosis of sepsis. In this study, many clinical predictors of prognosis of sepsis were identified. However, compared with the APACHE II score and lactic acid level, AFR had a weak predictive value for the prognosis of sepsis according to the ROC curve analysis. Therefore, we applied multiple factors to evaluate the predictive efficacy of prognosis to ensure reliability and accuracy. According to current data, AFR combined with the APACHE II score, lactic acid, and septic shock could increase the predictive value of prognosis compared with the APACHE II score, lactic acid, septic shock, and AFR alone in patients with sepsis. In other words, the combination of high baseline APACHE II score, high lactic acid level, the presence of septic shock, and low baseline AFR showed a higher risk of predicting 28-day mortality in patients with sepsis. To our knowledge, this is the first study to describe the combination of four predictive factors for the prognosis of sepsis, which may provide a novel direction for management of septic patients.

Several limitations of this study should be considered. First, this is a single-center, retrospective study with a relatively small sample size. Second, the clinical prognostic value of AFR is slight higher than albumin and fibrinogen in our study. More and further studies are needed. Last, it is unclear whether methods to improve the baseline AFR levels can significantly improve clinical prognosis.

## 5. Conclusion

Our study demonstrated that AFR was an independent predictor of 28-day mortality in patients with sepsis caused by various infection sites. AFR combined with APACHE II score, lactic acid, and septic shock showed a higher prognostic value for sepsis prognosis. However, prospective multicenter studies with larger sample sizes are needed to verify the prognostic value of AFR in patients experiencing sepsis.

## Figures and Tables

**Table 1:** Baseline characteristics of patients with sepsis between the survivor and non-survivor groups.

Parameters	Survivors (*n* = 153)	Nonsurvivors (*n* = 147)	*P* value
Age (years)	61.8 ± 15.6	64.1 ± 15.2	0.200
Gender, *n* (%)	—	—	0.089
Male	94 (61.4%)	104 (70.7%)	—
Female	59 (38.6%)	43 (29.3%)	—
Comorbidities	—	—	—
Hypertension, *n* (%)	28 (18.3%)	38 (25.9%)	0.115
Diabetes mellitus, *n* (%)	22 (14.4%)	30 (20.4%)	0.168
Chronic lung disease, *n* (%)	3 (2.0%)	10 (6.8%)	0.038^∗^
Cardiac disease, *n* (%)	12 (7.8%)	16 (10.9%)	0.365
Chronic renal disease, *n* (%)	9 (5.9%)	17 (11.6%)	0.080
Body temperature (°C)	37.1 ± 1.0	37.3 ± 1.2	0.124
Heart rate (per minute)	105.8 ± 24.5	116.0 ± 26.6	<0.001^∗^
Respiratory rate (per minute)	21.2 ± 6.0	24.7 ± 7.3	<0.001^∗^
Mean atrial pressure (mmHg)	79.4 ± 18.1	75.4 ± 20.9	0.007^∗^
Mechanical ventilation, n (%)	82 (53.6%)	105 (71.4%)	0.001^∗^
Continuous renal replacement therapy, *n* (%)	29 (19.0%)	41 (27.9%)	0.067
Septic shock, *n* (%)	111 (72.5%)	145 (98.6%)	<0.001^∗^
APACHE II score	15.9 ± 5.1	25.5 ± 7.7	<0.001^∗^
SOFA score	8.2 ± 4.0	12.3 ± 4.0	<0.001^∗^
GCS score	15.0 (*P*_25_ 12.0-*P*_75_15.0)	7.0 (*P*_25_ 3.0-*P*_75_14.0)	<0.001^∗^
Site of infection	—	—	0.004^∗^
Respiratory system, *n* (%)	32 (20.9%)	42 (28.6%)	—
Genitourinary system, *n* (%)	10 (6.5%)	12 (8.2%)	—
Gastrointestinal system, *n* (%)	30 (19.6%)	27 (18.3%)	—
Bloodstream, *n* (%)	22 (14.5%)	34 (23.1%)	—
Hepatobiliary system, *n* (%)	0 (0.0%)	2 (1.4%)	—
Central nervous system, *n* (%)	0 (0.0%)	2 (1.4%)	—
Unknown, *n* (%)	59 (38.5%)	28 (19%)	—

APACHE II: Acute Physiology and Chronic Health Evaluation; SOFA: Sequential Organ Failure Assessment; GCS: Glasgow Coma Scale; *P* values were calculated by chi-square test, Fisher's exact test, Student's *t*-test, or Mann–Whitney *U* test; ^∗^*P* < 0.05 indicates statistical significance.

**Table 2:** Laboratory variables of patients with sepsis between the survivor and nonsurvivor groups.

Parameters	Survivors (*n* = 153)	Nonsurvivors (*n* = 147)	*P* value
WBC (10^9^/L)	14.9 ± 9.0	14.6 ± 14.9	0.850
NEU (10^9^/L)	13.3 ± 8.4	12.9 ± 12.8	0.743
LYM (10^9^/L)	0.9 ± 1.8	0.9 ± 1.1	0.988
Hb (g/L)	105.0 ± 22.3	102.4 ± 30.1	0.400
PLTs (10^9^/L)	115.2 ± 85.3	101.6 ± 85.3	0.169
pH	7.5 ± 1.3	7.3 ± 0.1	<0.001^∗^
Lactic acid (mmol/L)	3.5 ± 2.2	6.0 ± 3.5	<0.001^∗^
CRP (mg/L)	151.4 ± 98.3	163.7 ± 97.7	0.280
PCT (ng/mL)	66.3 ± 41.8	60.9 ± 39.7	0.254
BUN (mmol/L)	13.3 ± 8.4	19.2 ± 13.9	<0.001^∗^
Creatinine (*μ*mol/L)	215.7 ± 183.7	278.1 ± 6.07	0.005^∗^
Albumin (g/L)	27.7 ± 5.6	25.0 ± 6.1	<0.001^∗^
Fibrinogen (g/L)	3.0 ± 1.2	4.0 ± 1.8	<0.001^∗^
AFR	11.0 ± 7.0	7.5 ± 3.6	<0.001^∗^

WBC: white blood cell; NEU: neutrophil; LYM: lymphocyte; Hb: hemoglobin; PLTs: platelets; CRP: C-reactive protein; PCT: procalcitonin; BUN: blood urea nitrogen; AFR: albumin-to-fibrinogen ratio; *P* values were calculated by Student's *t*-test or Mann–Whitney *U* test; ^∗^*P* < 0.05 indicates statistical significance.

**Table 3:** Risk factors for 28-day mortality in patients with sepsis by univariate and multivariate Cox proportional hazards regression analysis.

Variables	Univariable		Multivariable	
	HR (95% CI)	*P* value	HR (95% CI)	*P* value
Age	1.01 (0.99, 1.02)	0.200	1.01 (0.98, 1.03)	0.645
Gender (female)	0.66 (0.41, 1.07)	0.090	0.80 (0.35, 1.80)	0.588
Chronic lung disease (yes vs. no)	3.68 (0.99, 13.64)	0.052	5.55 (0.66, 46.59)	0.114
Heart rate (per minute)	1.02 (1.01, 1.03)	<0.001^∗^	0.99 (0.98, 1.01)	0.512
Respiratory rate (per minute)	1.08 (1.05, 1.13)	<0.001^∗^	1.01 (0.95, 1.08)	0.768
Mean atrial pressure (mmHg)	0.99 (0.98, 1.00)	0.082	1.00 (0.98, 1.02)	0.732
Mechanical ventilation (yes vs. no)	2.16 (1.34, 3.49)	0.002^∗^	1.75 (0.80, 3.82)	0.162
Septic shock (yes vs. no)	27.43 (6.50, 115.76)	<0.001^∗^	21.44 (2.51, 182.76)	0.005^∗^
APACHE II score	1.25 (1.19, 1.32)	<0.001^∗^	1.18 (1.07, 1.30)	<0.001^∗^
SOFA score	1.28 (1.19, 1.36)	<0.001^∗^	1.03 (0.91, 1.16)	0.616
GCS score	0.75 (0.70, 0.81)	<0.001^∗^	0.92 (0.81, 1.05)	0.197
Site of infection	—	—	—	—
Respiratory infection (yes vs. no)	1.0 (reference)	—	1.0 (reference)	—
Genitourinary infection (yes vs. no)	0.91 (0.35, 2.38)	0.854	—	—
Gastrointestinal infection (yes vs. no)	0.69 (0.34, 1.37)	0.287	—	—
Bloodstream infection (yes vs. no)	1.18 (0.58, 2.39)	0.650	—	—
Hepatobiliary infection (yes vs. no)	4386905 (0, ∞)	0.988	—	—
Central nervous infection (yes vs. no)	4386905 (0, ∞)	0.988	—	—
Unknown (yes vs. no)	0.36 (0.19,0.69)	0.002^∗^	0.81 (0.26, 2.51)	0.708
pH	0.004 (0.001, 0.029)	<0.001^∗^	0.35 (0.01, 11.65)	0.556
Lactic acid (mmol/L)	1.42 (1.28, 1.58)	<0.001^∗^	1.27 (1.08, 1.50)	0.005^∗^
BUN (mmol/L)	1.06 (1.03, 1.09)	<0.001^∗^	1.04 (0.98, 1.09)	0.163
Creatinine (*μ*mol/L)	1.002 (1.000, 1.003)	0.007^∗^	1.00 (0.99, 1.0)	0.068
Albumin (g/L)	0.92 (0.88, 0.96)	<0.001^∗^	—	—
Fibrinogen (g/L)	1.52 (1.29, 1.80)	<0.001^∗^	—	—
AFR	0.84 (0.79, 0.90)	<0.001^∗^	0.70 (0.62, 0.80)	<0.001^∗^

APACHE II: Acute Physiology and Chronic Health Evaluation; SOFA: Sequential Organ Failure Assessment; GCS: Glasgow Coma Scale; BUN: blood urea nitrogen; AFR: albumin-to-fibrinogen ratio; HR: hazard ratio; CI: confidence interval; ^∗^*P* < 0.05 indicates statistical significance.

**Table 4:** Predictive valve of APACHE II score, lactic acid, septic shock, AFR, triple index, and quadruple index for 28-day mortality in patients with sepsis.

Variables	ROC area (AUC)	95% CI	Best threshold	Specificity	Sensitivity
APACHE II score	0.849	0.806~0.892	21.50	0.869	0.687
Lactic acid (mmol/L)	0.741	0.683~0.798	4.095	0.745	0.701
Septic shock	0.631	0.594~0.667	NA	0.275	0.986
AFR	0.700	0.641~0.758	5.860	0.908	0.421
Triple index	0.877	0.839~0.915	-0.412	0.778	0.823
Quadruple index	0.922	0.893~0.951	0.0024	0.869	0.836

ROC: receiver operating characteristic; AUC: area under the curve; APACHE II: Acute Physiology and Chronic Health Evaluation; AFR: albumin-to-fibrinogen ratio; Triple index includes APACHE II score, lactic acid, and septic shock; quadruple index includes APACHE II score, lactic acid, septic shock, and AFR; CI: confidence interval.

## Data Availability

The raw data supporting the conclusions of this article will be made available by the authors, without undue reservation.
